# Physiological Traits for Climate‐Ready Restoration

**DOI:** 10.1002/ece3.72939

**Published:** 2026-01-12

**Authors:** Kasey E. Barton, Cate Macinnis‐Ng, Rebecca Ostertag, Aurora Kagawa‐Viviani

**Affiliations:** ^1^ School of Life Sciences University of Hawaiʻi at Mānoa Honolulu Hawaiʻi USA; ^2^ School of Biological Sciences Waipapa Taumata Rau the University of Auckland Auckland New Zealand; ^3^ Department of Biology University of Hawaiʻi at Hilo Hilo Hawaiʻi USA; ^4^ Water Resources Research Center and Department of Geography and Environment University of Hawaiʻi at Mānoa Honolulu Hawaiʻi USA

**Keywords:** co‐production, drought, ecophysiology, functional traits, outplanting, phenotypic plasticity

## Abstract

Trait‐based approaches feature prominently in ecology, providing mechanistic insights into plant interactions with abiotic and biotic factors. Increasingly, traits are used in restoration and conservation to select species with target functions and to monitor performance in restoration settings. Physiological traits are rarely used, despite their clear relevance for restoration in the context of climate change. We provide rationale and guidance for the expanded integration of physiological traits (e.g., metabolic and energy transfer processes, gas exchange, nutrient dynamics, and hydraulics) into restoration to enhance resilience of terrestrial ecosystems to climate change. We identify traits mediating the tolerance of climate stressors, particularly drought, heat, and salinity due to sea level rise. Physiological traits are highly dynamic, within and among species, and this variability can be leveraged to inform species selection and monitoring efforts. Trait variability via phenotypic plasticity and climate stress thresholds offers key insights into plant performance and population stability under climate stress. Trait‐based approaches complement existing restoration and conservation strategies, including those based on Indigenous ecological knowledge and resource management. Effective researcher‐practitioner collaborations are essential to successfully integrate physiology into ecological restoration. *Synthesis and applications*. Climate stress is increasing globally, and physiological traits are critical for understanding stress tolerance in plants. Integrating physiology into restoration will contribute to climate resilience, but depends on partnerships between researchers and practitioners and will benefit from increased physiological trait sampling of more species, sites, and traits. Expanded trait coverage will enhance species selection and performance monitoring, improving restoration and species stabilization in a changing climate.

## Graphical abstract

Plant physiological traits can offer key insights into plant performance under climate change, calling for their increased use to inform restoration via species selection and monitoring. Increased collaboration between restoration practitioners and physiologist researchers provide the means by which this approach can be realized.

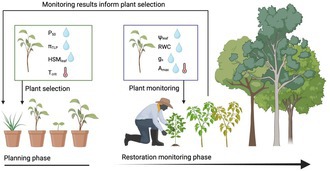

## 1 Introduction

Restoration activities are designed to meet diverse goals, commonly including native biodiversity, species complementarity, invasion resistance, ecosystem function and stability, food provisioning, and cultural services (Hallett et al. 2013). As a complement to traditional and Indigenous approaches, trait‐based approaches are emerging as useful for species selection and monitoring of plants in restoration (Loureiro et al. 2023). Functional traits, defined as physiological, anatomical, morphological, phenological, and architectural traits, are of particular interest because of their links to fitness via effects on survival, growth, and reproduction (Violle et al. 2007), and because they underlie plant effects on ecosystem processes such as nutrient cycling, carbon sequestration, and water balance (Lavorel and Garnier 2002). For example, community‐scale productivity is better predicted by functional traits than taxonomy for restored assemblages in a California grassland (Funk et al. 2023). In a tropical wet forest restoration experiment in lowland Hawaiʻi, assemblies of species with diverse functional traits are more resistant to invasion than assemblages with lower functional diversity (DiManno et al. 2023). As climate change resilience becomes an increasingly common conservation goal (Prober et al. 2019; Buenafe et al. 2025; Simonson et al. 2021), traits that predict climate stress tolerance will be particularly informative. We argue here that physiological traits offer promising insights to advance trait‐based restoration with the goal of promoting climate‐ready and climate‐resilient conservation.

Implementing a physiological trait‐based approach for restoration will require new levels of partnership and collaboration among researchers and natural resource practitioners. Given the significant cost of restoration failure and the likely increase in the incidence of failure under climate change, investment in collaborative efforts through time, funding, and capacity building will ultimately become more economical through a reduction in restoration failures. Therefore, investing now in the expansion of physiological trait‐based restoration will provide a cost‐savings in the long term. To achieve this goal, researchers and practitioners must work together to co‐produce projects that entail physiological trait analysis embedded within restoration actions. Physiologists often lack the local knowledge needed to identify the species of greatest conservation and restoration value as well as the climate threats for which trait data are most informative, which practitioners can provide. Conversely, many practitioners lack the time, instrumentation, and specialized knowledge required to measure and interpret physiological traits, although a limited set of traits can be measured using simple handheld instruments, such as porometers and fluorometers (Cooke and Suski 2008). Despite historical disconnects between researcher‐practitioner goals and constraints (Merchant et al. 2022; Gornish et al. 2023), we propose a path for applying co‐produced research (Nel et al. 2016) to enhance restoration and conservation activities under climate change. Specifically, practitioners will first identify the restoration focus—selecting the site and potential species of interest, as well as the likely climate stress factors most limiting restoration success. Physiologists bring the expertise to suggest the most informative traits to measure as predictors of plant performance under stress. Together, practitioners and researchers will develop the scope of trait sampling needed to inform the restoration project; this may include a priori experimentation and surveying to delineate ranges of optimal trait expression, thresholds of shifting trait expression under climate stress, and the role of phenotypic plasticity and population differentiation to inform species selection. Trait sampling, analysis, and interpretation will involve ongoing interactions between researchers and practitioners to ensure immediate implementation and adaptive methods to reflect practitioner concerns. There is tremendous diversity in plants, their strategies to tolerate climate stress, as well as diversity in practitioner priorities, making a single plan to fit all scenarios impossible. Below, we provide additional discussion of how physiological traits can inform climate stress tolerance to inform species selection and performance monitoring in restoration, including a partial list of informative physiological traits (Table 1). However, we emphasize the need for interactions between researchers and practitioners early in the planning stages of restoration to accommodate the specifics of a particular project.

**TABLE 1:** Key plant physiological traits relevant to plant productivity and water relations that are linked to climate stress tolerance, and which may be useful to inform restoration plant selection (PS) and have value for monitoring restoration success (restoration monitoring: RM). Climate stressor categories are heat (thermometer), water (water drop), and salinity (salt shaker). A stress category in brackets indicates a secondary response. This list is not exhaustive, and physiologist‐practitioner teams may identify other traits associated with alternative climate stress tolerance strategies to focus their efforts on.

Variable and abbreviation	Information provided	Stressor	Instrumentation	Accessibility	Accessibility	Primary application
		Heat/water/salinity		Cost	Level of expertise	Plant selection/restoration monitoring
Photosynthetic heat tolerance (*T*_50_ and *T*_crit_)	Temperature at which photosystems begin to decline (*T*_crit_) and the temperature at which 50% of photosystems are damaged (*T*_50_).	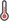	Chlorophyll fluorometer or conductivity meter for electrolyte leakage	$$/$$$	Moderate–high	PS
Chlorophyll *a* fluorescence (Δ*F*/*Fm*′)	Indicates photosynthetic efficiency which declines under light, heat and water stress.	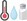	Fluorometer	$$/$$$	high	RM
Biochemical constraints of photosynthesis (*V*_cmax_ and *J*_max_)	*V*_cmax_ is the maximum rate of Rubisco carboxylase activity and J_max_ is maximum electron transport rate. Both are temperature‐dependent. Higher values indicate faster photosynthesis for better performance.	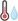	Leaf gas exchange system used to derive A/C_i_ curves or “one point” method	$$$	High	PS
Carbon isotope signature (δ^13^C)	Integrated water use efficiency of leaf, plant or ecosystem. Plants with improved WUE should be less vulnerable to drought	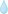	Mass spectrometry for carbon isotope analysis	$$$	high	PS/RM
Assimilation rate (*A*_max_)	Rate of photosynthetic carbon uptake. Will likely decrease under water, heat and salt stress	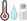	Leaf gas exchange system	$$$	high	RM
Chlorophyll pigments	Indicates concentration of chlorophyll and accessory pigments. Decrease in chlorophyll (or increase in accessory pigments) may be associated with stress due to heat or light.	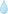	Hand‐held analyzer or laboratory extraction	$$	Low	RM
Vulnerability to xylem embolism (P_50_)	Water potential at which 50% of hydraulic conductance is lost. Species with a low P_50_ are more vulnerable to xylem embolism and therefore more vulnerable to drought.	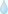	Pressure chamber and hydraulic apparatus	$$	High	PS
Turgor loss point and associated variables (TLP, *π*_tlp_)	Water potential at which plants lose turgor. Plants with more negative TLP are less vulnerable to drought.	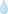	Pressure chamber	$$	High	PS
Leaf water potential (Ψ_leaf_)	Indicates water status of plants with more negative values associated with development of water stress.	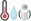	Pressure chamber	$$	Moderate	RM
Relative water content (RWC)	Tissue water content in leaves, stems, roots. Indicates desiccation of plant tissues—particularly useful in drought.	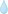	High precision balance, drying oven	$	Low	RM
Hydraulic safety margin (HSM_leaf_)	Difference between P_50_ and minimum water potential under natural drought conditions. Plants with a larger safety margin are less vulnerable to drought.	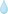	Pressure chamber and hydraulic apparatus	$$	High	PS
Stem capacitance (*C*_s_)	Amount of stored water in stems. Plants with more stored water are less vulnerable to drought.	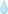	Bench water release curves or sap flow methods	$$	High	PS
Stomatal conductance (*g*_s_)	Rate of water vapor diffusion through stomata. Declines when stomata close, especially in response to declining water availability in soil and air	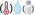	Leaf gas exchange system or porometer	$$	High	RM

## 2 Physiological Trait‐Based Approaches

Trait‐based restoration currently relies predominantly on morphological traits, reflecting their ease of measurement and widespread availability in trait databases (Kattge et al. 2020). For example, among the 30 traits with the best species coverage in the TRY database (Kattge et al. 2020), most are categorical and only two are physiological (leaf nitrogen content and stem specific density). Because of this limited availability, trait‐based restoration tools include relatively few archived physiological trait data (Rayome et al. 2019), or they require users to measure the trait data themselves (Wang et al. 2021), which is impractical for many practitioners. As climate change accelerates, traits that predict plant survival, growth, and reproduction under climate stress will be the most useful. Physiological traits are more directly related to climate stress tolerance than morphological traits are, and so greater integration of physiological traits into restoration and conservation activities will help achieve climate‐ready restoration goals (Martinez‐Garza et al. 2013). For example, among a diverse suite of physiological and morphological traits, only foliar δ^13^C measured during the dry season accurately predicted two‐year survival rates across 32 species in a tropical dry forest experiment in Costa Rica (Werden et al. 2018).

Functional traits are measured at the scale of individual plants, but species‐level trait means are often used in trait‐based restoration. Although this practice follows from the general pattern that trait variability is greater among than within species (Siefert et al. 2015), there are clear benefits to considering intraspecific variation in physiological traits for restoration (Cooke and Suski 2008). Intraspecific trait variation (ITV) is widespread and common, of considerable magnitudes, and generally of greater importance for physiological than morphological traits (Siefert et al. 2015; Westerband et al. 2021), emphasizing its particular relevance for climate‐ready restoration. ITV occurs over space and time, reflecting local adaptation to heterogeneous environments, as well as intra‐individual variability via phenotypic plasticity and ontogeny (Barton 2024; Westerband et al. 2021), and both spatial and temporal dimensions of ITV are relevant to meet the goals of species selection and performance monitoring under climate change. Because physiological trait sampling and interpretation require specialized instruments and expertise, collaboration between researchers and practitioners is the most promising way to achieve this goal. Our aim here is to provide guidance for the increased use of physiological traits for climate‐ready restoration by identifying physiological traits to inform species selection and monitoring and by describing scenarios for productive collaboration between researchers and practitioners.

## 3 Climate Change and Plant Physiology

Climate change is now recognized as a key aspect to plan for in restoration (Simonson et al. 2021). Climate stress of some kind is a feature of most habitats, and plants have evolved diverse strategies to tolerate drought, freezing temperatures, and heat waves. Nonetheless, the magnitude and frequency of climate stress under global change will, or in some cases, already do exceed historical patterns (Masson‐Delmotte et al. 2021), driving increased mortality and stand‐level dieback in plants (Allen et al. 2010). In addition to intensified or more frequently occurring temperature and water‐related climate extremes, sea level rise is increasing substrate salinity in coastal regions, and so we consider salinity stress to be a component of climate change. Water, temperature, and salinity (i.e., ion balance) are fundamental factors influencing metabolic processes and homeostasis, underscoring the importance of physiology in plant resilience to climate change. These factors are also interdependent, making it difficult to determine the causal links between climate change and plant declines. For example, drought and heat interact in their effects on plants because drought‐induced stomatal closure reduces transpiration as a strategy to conserve water, but consequently causes heat stress due to the lack of transpirational cooling (Chaves et al. 2016). Salinity and water stress are similarly linked; elevated salinity causes direct ion toxicity but also lowers soil water potential, limiting root water uptake (Sheldon et al. 2017), a dynamic that is further exacerbated by drought.

**FIGURE 1:**
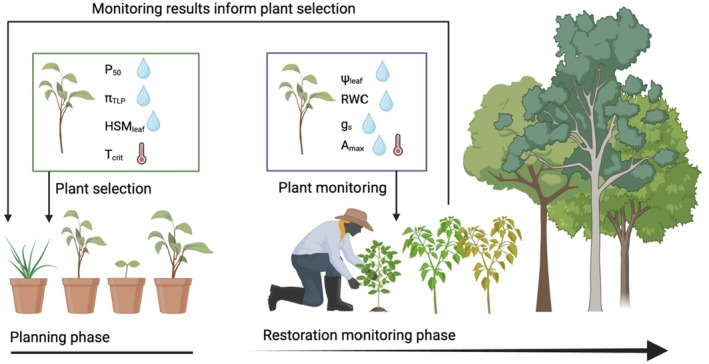
Examples of traits that can be used in plant selection (left) and plant monitoring (right). Water drops indicate traits for assessing water stress and thermometer symbols indicate traits for heat stress. Selection traits include vulnerability to xylem embolism (P_50_), water potential at turgor loss point (Π_TLP_), leaf hydraulic safety margin (HSM_leaf_), and temperature of photosystem damage (*T*_crit_), see Table 1 for more details. Monitoring traits include leaf water potential (Ψ_leaf_), relative water content (RWC), stomatal conductance (*g*_s_), and maximum assimilation rate (*A*_max_). Monitoring traits can be tracked through time (for instance, as a drought progresses) or compared to reference traits (for instance, Ψ_leaf_ may be compared to P_50_ to produce hydraulic safety margin). Monitoring data can also be used to inform plant selection in future restoration projects.

Plant physiologists have investigated plant strategies underlying climate stress extensively, and functional traits that are key indicators of stress‐tolerant plants have emerged that could be targeted to inform restoration under climate change (Table 1). These physiological traits can be used in restoration in two primary ways—as the basis for selecting species with known climate stress tolerance, and as the basis for monitoring plant performance during a climate stress event or ongoing baseline climatic change (Figure 1). Often, different traits are informative for these two approaches. Here we provide guidance on traits that are well‐documented for climate stress tolerance, and which will be useful to consider for restoration under three common and increasing climate stressors, but this is not an exhaustive list given the diversity of climate stress tolerance strategies found among species (Table 1).

1. Drought—Across biomes, drought resistance has been linked to low leaf turgor loss point and resistance to embolism (captured via leaf water potential at 50% loss stem hydraulic conductance, referred to as P_50_), as well as the associated anatomical trait, high wood density (O'Brien et al. 2017; Anderegg 2015; Bartlett et al. 2012).
2. Salinity—Salinity tolerance is achieved via diverse strategies of ion exclusion, sequestration, or exudation, and is often linked with tolerance to water limitation as salinity and water stress commonly co‐occur (Munns 2002). Physiological traits associated with salinity tolerance include low root water potential, high chlorophyll content and water use efficiency, rapid stomatal regulation, high levels of ion storage in various leaf or root tissues, and succulence (Lum and Barton 2020; Munns and Tester 2008; Munns 2002).
3. Heat—Heat stress affects photosynthesis, respiration, water relations, membrane stability, and can also influence primary and secondary metabolites (Wahid et al. 2007). Thermotolerance mechanisms include the production of stress‐related proteins and reactive oxygen species to counteract impact on photosynthesis and improve membrane stability (Hemantaranjan et al. 2018), and a range of other biochemical and transcriptional adjustments to mitigate the impacts of heat (Wahid et al. 2007). Traits frequently used to measure heat stress are often related to photosynthesis, such as the critical temperature at which photosystem II is disrupted: *T*_crit_ (O'Sullivan et al. 2017).

A fourth increasing climate stressor is flooding, and although many species can survive short periods of flooding (Jackson and Colmer 2005), there are few functional trait data measured in response to such intermittent and irregular flooding. Species adapted to chronic inundation (such as mangroves) are well studied, and tolerance usually involves specialized anatomical adaptations such as aerenchyma tissue in roots to enhance aeration or thinner leaf cuticles and cell walls in submerged leaves to reduce resistance to diffusion of CO_2_ into the leaf (Voesenek and Bailey‐Serres 2015). Because of the limited data for nonadapted species experiencing infrequent inundation, we exclude this climate stressor from further consideration and identify it as an area needing further research.

## 4 Physiological Traits for Species Selection

Physiological trait data provide the basis to predict which species will persist or decline as climate stress amplifies, offering a pathway for climate‐ready restoration. For example, species with high embolism resistance and low leaf turgor loss point can be outplanted in sites where drought intensity and frequency are increasing, while sensitive species with low embolism resistance and high leaf turgor loss point can be outplanted in sites where precipitation is increasing or prioritized for additional management to mitigate drought stress in dry sites (Figure 1). Similarly, species with excretory salt glands and high leaf water‐use efficiency are likely to be robust to increased salinity and so will be critical for beach stabilization under sea level rise. Prioritizing sampling of these key indicators of climate stress tolerance across more species at more sites, including across populations within species spanning climate gradients, is essential to inform trait‐based restoration decisions.

For many species, there is evidence that physiological ITV among populations corresponds to local adaptation. For example, P_50_ varies considerably within species and is lower in populations at xeric sites (Anderegg 2015), reflecting adaptation to water stress. Although provenance trials have already documented plant population variability in fitness metrics (e.g., growth) relevant to climate change (Prober et al. 2015), these trials rarely investigate physiological traits for a mechanistic understanding of local adaptation. Measuring physiological traits in provenance trials could elucidate physiological traits that are markers of climate stress‐tolerant populations, which could then be used to screen additional populations without the intensive garden experiments typical of provenance trials. For species predicted to face a loss of habitat due to climate change (Fortini et al. 2013), physiological ITV can facilitate conservation actions such as assisted gene flow and assisted migration to match plant populations with the climates to which they are adapted (Aitken and Whitlock 2013).

## 5 Physiological Traits to Monitor Plant Performance

In addition to species selection, monitoring plant physiological traits during periods of climate stress can be critical for detecting declines in plant performance before lethal thresholds are reached (Figure 1). For this approach to be useful, an a priori understanding of species physiological function is needed, requiring experimental or field‐based sampling during nonstressed and stressed conditions to characterize the dynamic of trait plasticity associated with climate stress tolerance. In particular, three kinds of physiological trait expression and plasticity are relevant to restoration. First, pre‐stress (i.e., basal or constitutive) trait expression can underlie stress tolerance by predisposing plants to sensitivity. For example, low leaf water potentials in xeric plants (Liu et al. 2003) and pre‐winter production of plant antifreeze compounds (Preston and Sandve 2013) are examples of constitutively expressed climate stress physiological traits. Second, many plants express shifts in trait expression in response to stress via phenotypic plasticity (Matesanz et al. 2010), and it is this plasticity that determines stress tolerance. Reduced stomatal conductance via stomatal regulation in response to drought and salinity (Lum and Barton 2020; Westerband et al. 2019; Ravi et al. 2021), acclimation of Rubisco activity and electron transport rates during drought (Zhou et al. 2015), upregulation of heat shock proteins (Hemantaranjan et al. 2018), and ethylene release to stimulate aerenchyma in flooded roots (Voesenek and Bailey‐Serres 2015) illustrate the importance of physiological trait plasticity in climate stress tolerance. Third, physiological traits expressed as thresholds are commonly used to assess climate stress vulnerability. For example, P_50_ (and related thresholds, such as P_15_ and P_80_) provide the basis for comparison across species in vulnerability to embolisms (Anderegg et al. 2016). Similarly, *T*_50_ refers to the temperature at which 50% leaf damage occurs, providing a threshold of heat damage (Doughty et al. 2023). Such threshold metrics provide reference points for comparison with monitored plant responses to ongoing climate stress (Schönbeck et al. 2023), and can also be used as a basis for species selection (Table 1).

## 6 Conclusions and Future Directions

Climate change poses urgent threats to biological diversity, and tools that promote climate‐ready restoration are becoming increasingly important. Physiological traits offer promising insights to enhance climate‐ready restoration, but their implementation seems overwhelmingly difficult due to a lack of existing, available data, access to instrumentation, and expertise. Training students in physiological trait measurement and interpretation is needed to develop a conservation workforce with the skills to implement physiological trait‐based approaches. Finally, none of this is feasible without the development of enduring researcher‐practitioner relationships to facilitate knowledge transfer and co‐production of research to inform climate‐ready conservation.

## Author Contributions

**Kasey E. Barton:** conceptualization (lead), writing – original draft (lead), writing – review and editing (lead). **Cate Macinnis‐Ng:** conceptualization (equal), writing – original draft (equal), writing – review and editing (supporting). **Rebecca Ostertag:** conceptualization (supporting), writing – review and editing (supporting). **Aurora Kagawa‐Viviani:** conceptualization (supporting), writing – review and editing (supporting).

## Funding

This work was supported by the U.S. National Science Foundation (grant numbers 2218916 and OIA‐2149133).

## Conflicts of Interest

The authors declare no conflicts of interest.

## Data Availability

No data were collected or analyzed in this paper.

## References

[ece372939-bib-0001] Aitken, S. N. , and M. C. Whitlock . 2013. “Assisted Gene Flow to Facilitate Local Adaptation to Climate Change.” Annual Review of Ecology, Evolution, and Systematics 44: 367–388. 10.1146/annurev-ecolsys-110512-135747.

[ece372939-bib-0002] Allen, C. D. , A. K. Macalady , H. Chenchouni , et al. 2010. “A Global Overview of Drought and Heat‐Induced Tree Mortality Reveals Emerging Climate Change Risks for Forests.” Forest Ecology and Management 259: 660–684. 10.1016/j.foreco.2009.09.001.

[ece372939-bib-0003] Anderegg, W. R. L. 2015. “Spatial and Temporal Variation in Plant Hydraulic Traits and Their Relevance for Climate Change Impacts on Vegetation.” New Phytologist 205: 1008–1014. 10.1111/nph.12907. 25729797

[ece372939-bib-0004] Anderegg, W. R. L. , T. Klein , M. Bartlett , et al. 2016. “Meta‐Analysis Reveals That Hydraulic Traits Explain Cross‐Species Patterns of Drought‐Induced Tree Mortality Across the Globe.” Proceedings of the National Academy of Sciences of the United States of America 113: 5024–5029. 10.1073/pnas.1525678113. 27091965 PMC4983847

[ece372939-bib-0005] Bartlett, M. K. , C. Scoffoni , and L. Sack . 2012. “The Determinants of Leaf Turgor Loss Point and Prediction of Drought Tolerance of Species and Biomes: A Global Meta‐Analysis.” Ecology Letters 15: 393–405. 10.1111/j.1461-0248.2012.01751.x. 22435987

[ece372939-bib-0006] Barton, K. E. 2024. “The Ontogenetic Dimension of Plant Functional Ecology.” Functional Ecology 38: 98–113. 10.1111/1365-2435.14464.

[ece372939-bib-0007] Buenafe, K. C. V. , D. C. Dunn , A. Metaxas , et al. 2025. “Current Approaches and Future Opportunities for Climate‐Smart Protected Areas.” Nature Reviews Biodiversity 1: 284–297. 10.1038/s44358-025-00041-0.

[ece372939-bib-0008] Chaves, M. M. , J. M. Costa , O. Zarrouk , C. Pinheiro , C. M. Lopes , and J. S. Pereira . 2016. “Controlling Stomatal Aperture in Semi‐Arid Regions—The Dilemma of Saving Water or Being Cool?” Plant Science 251: 54–64. 10.1016/j.plantsci.2016.06.015. 27593463

[ece372939-bib-0009] Cooke, S. J. , and C. D. Suski . 2008. “Ecological Restoration and Physiology: An Overdue Integration.” Bioscience 58: 957–968.

[ece372939-bib-0010] DiManno, N. , R. Ostertag , A. Uowolo , et al. 2023. “Functional Trait‐Based Restoration Alters Nutrient Cycling and Invasion Rates in Hawaiian Lowland Wet Forest.” Ecological Applications 33: 14. 10.1002/eap.2894. 37282355

[ece372939-bib-0011] Doughty, C. E. , J. M. Keany , B. C. Wiebe , et al. 2023. “Tropical Forests Are Approaching Critical Temperature Thresholds.” Nature 621: 105–111. 10.1038/s41586-023-06391-z. 37612501

[ece372939-bib-0012] Fortini, L. , J. Price , J. Jacobi , et al. 2013. “A Landscape‐Based Assessment of Climate Change Vulnerability for All Native Hawaiian Plants.” USGS Technical Report, pp. 141.

[ece372939-bib-0013] Funk, J. L. , S. Kimball , M. A. Nguyen , M. Lulow , and G. E. Vose . 2023. “Interacting Ecological Filters Influence Success and Functional Composition in Restored Plant Communities Over Time.” Ecological Applications 33: 16. 10.1002/eap.2899. 37335271

[ece372939-bib-0014] Gornish, E. S. , C. Campbell , L. Svejcar , et al. 2023. “Functional Traits Are Used in Restoration Practice: A Response to Merchant Et al. (2022).” Restoration Ecology 31: e13880. 10.1111/rec.13880.

[ece372939-bib-0015] Hallett, L. M. , S. Diver , M. V. Eitzel , et al. 2013. “Do we Practice What We Preach? Goal Setting for Ecological Restoration.” Restoration Ecology 21: 312–319. 10.1111/rec.12007.

[ece372939-bib-0016] Hemantaranjan, A. , C. Malik , and A. N. Bhanu . 2018. “Physiology of Heat Stress and Tolerance Mechanisms—An Overview.” Journal of Plant Science Research 33: 55–68.

[ece372939-bib-0017] Jackson, M. B. , and T. D. Colmer . 2005. “Response and Adaptation by Plants to Flooding Stress.” Annals of Botany 96: 501–505. 10.1093/aob/mci205. 16217870 PMC4247020

[ece372939-bib-0018] Kattge, J. , G. Bönisch , S. Díaz , et al. 2020. “TRY Plant Trait Database – Enhanced Coverage and Open Access.” Global Change Biology 26: 119–188. 10.1111/gcb.14904. 31891233

[ece372939-bib-0019] Lavorel, S. , and E. Garnier . 2002. “Predicting Changes in Community Composition and Ecosystem Functioning From Plant Traits: Revisiting the Holy Grail.” Functional Ecology 16: 545–556. 10.1046/j.1365-2435.2002.00664.x.

[ece372939-bib-0020] Liu, M. Z. , G. M. Jiang , Y. G. Li , et al. 2003. “Leaf Osmotic Potentials of 104 Plant Species in Relation to Habitats and Plant Functional Types in Hunshandak Sandland, Inner Mongolia, China.” Trees 17: 554–560. 10.1007/s00468-003-0277-8.

[ece372939-bib-0021] Loureiro, N. , D. Mantuano , A. Manhas , and J. Sansevero . 2023. “Use of the Trait‐Based Approach in Ecological Restoration Studies: A Global Review.” Trees 37: 1287–1297. 10.1007/s00468-023-02439-9.

[ece372939-bib-0022] Lum, T. D. , and K. E. Barton . 2020. “Ontogenetic Variation in Salinity Tolerance and Ecophysiology of Coastal Dune Plants.” Annals of Botany 125: 301–314. 10.1093/aob/mcz097. 31162531 PMC7442332

[ece372939-bib-0023] Martinez‐Garza, C. , F. Bongers , and L. Poorter . 2013. “Are Functional Traits Good Predictors of Species Performance in Restoration Plantings in Tropical Abandoned Pastures?” Forest Ecology and Management 303: 35–45. 10.1016/j.foreco.2013.03.046.

[ece372939-bib-0024] Masson‐Delmotte, V. , P. Zhai , A. Pirani , et al. 2021. IPCC, 2021: Climate Change 2021: The Physical Science Basis. Contribution of Working Group I to the Sixth Assessment Report of the Intergovernmental Panel on Climate Change. Cambridge University Press.

[ece372939-bib-0025] Matesanz, S. , E. Gianoli , and F. Valladares . 2010. “Global Change and the Evolution of Phenotypic Plasticity in Plants.” Annals of the New York Academy of Sciences 1206: 35–55. 10.1111/j.1749-6632.2010.05704.x. 20860682

[ece372939-bib-0026] Merchant, T. K. , J. J. Henn , I. de Silva , E. Van Cleemput , and K. N. Suding . 2022. “Four Reasons Why Functional Traits Are Not Being Used in Restoration Practice.” Restoration Ecology 31: e13788. 10.1111/rec.13788.

[ece372939-bib-0027] Munns, R. 2002. “Comparative Physiology of Salt and Water Stress.” Plant, Cell & Environment 25: 239–250. 10.1046/j.0016-8025.2001.00808.x. 11841667

[ece372939-bib-0028] Munns, R. , and M. Tester . 2008. “Mechanisms of Salinity Tolerance.” Annual Review of Plant Biology 59: 651–681. 10.1146/annurev.arplant.59.032607.092911 18444910

[ece372939-bib-0029] Nel, J. L. , D. J. Roux , A. Driver , et al. 2016. “Knowledge Co‐Production and Boundary Work to Promote Implementation of Conservation Plans.” Conservation Biology 30: 176–188. 10.1111/cobi.12560. 26041340

[ece372939-bib-0030] O'Brien, M. J. , B. M. J. Engelbrecht , J. Joswig , et al. 2017. “A Synthesis of Tree Functional Traits Related to Drought‐Induced Mortality in Forests Across Climatic Zones.” Journal of Applied Ecology 54: 1669–1686. 10.1111/1365-2664.12874.

[ece372939-bib-0031] O'Sullivan, O. S. , M. A. Heskel , P. B. Reich , et al. 2017. “Thermal Limits of Leaf Metabolism Across Biomes.” Global Change Biology 23: 209–223. 10.1111/gcb.13477. 27562605

[ece372939-bib-0032] Preston, J. C. , and S. R. Sandve . 2013. “Adaptation to Seasonality and the Winter Freeze.” Frontiers in Plant Science 4: 167. 10.3389/fpls.2013.00167. 23761798 PMC3669742

[ece372939-bib-0033] Prober, S. M. , M. Byrne , E. H. McLean , et al. 2015. “Climate‐Adjusted Provenancing: A Strategy for Climate‐Resilient Ecological Restoration.” Frontiers in Ecology and Evolution 3: 65. 10.3389/fevo.2015.00065.

[ece372939-bib-0034] Prober, S. M. , V. A. J. Doerr , L. M. Broadhurst , K. J. Williams , and F. Dickson . 2019. “Shifting the Conservation Paradigm: A Synthesis of Options for Renovating Nature Under Climate Change.” Ecological Monographs 89: 23. 10.1002/ecm.1333.

[ece372939-bib-0035] Ravi, S. , M. K.‐F. Bader , T. Young , et al. 2021. “Are the Well‐Fed Less Thirsty? Effects of Drought and Salinity on New Zealand Mangroves.” Journal of Plant Ecology 15: 85–99. 10.1093/jpe/rtab071.

[ece372939-bib-0036] Rayome, D. , N. DiManno , R. Ostertag , et al. 2019. “Restoring Ecosystem Services Tool (REST): A Program for Selecting Species for Restoration Projects Using a Functional‐Trait Approach.” U.S. Department of Agriculture, Forest Service, Pacific Southwest Research Station.

[ece372939-bib-0037] Schönbeck, L. , M. Arteaga , H. Mirza , et al. 2023. “Plant Physiological Indicators for Optimizing Conservation Outcomes.” Conservation Physiology 11: coad073. 10.1093/conphys/coad073. 37711583 PMC10498484

[ece372939-bib-0038] Sheldon, A. R. , R. C. Dalal , G. Kirchhof , P. M. Kopittke , and N. W. Menzies . 2017. “The Effect of Salinity on Plant‐Available Water.” Plant and Soil 418: 477–491. 10.1007/s11104-017-3309-7.

[ece372939-bib-0039] Siefert, A. , C. Violle , L. Chalmandrier , et al. 2015. “A Global Meta‐Analysis of the Relative Extent of Intraspecific Trait Variation in Plant Communities.” Ecology Letters 18: 1406–1419. 10.1111/ele.12508. 26415616

[ece372939-bib-0040] Simonson, W. D. , E. Miller , A. Jones , S. García‐Rangel , H. Thornton , and C. McOwen . 2021. “Enhancing Climate Change Resilience of Ecological Restoration — A Framework for Action.” Perspectives in Ecology and Conservation 19: 300–310. 10.1016/j.pecon.2021.05.002.

[ece372939-bib-0041] Violle, C. , M. L. Navas , D. Vile , et al. 2007. “Let the Concept of Trait Be Functional!” Oikos 116: 882–892. 10.1111/j.2007.0030-1299.15559.x.

[ece372939-bib-0042] Voesenek, L. A. C. J. , and J. Bailey‐Serres . 2015. “Flood Adaptive Traits and Processes: An Overview.” New Phytologist 206: 57–73. 10.1111/nph.13209. 25580769

[ece372939-bib-0043] Wahid, A. , S. Gelani , M. Ashraf , and M. R. Foolad . 2007. “Heat Tolerance in Plants: An Overview.” Environmental and Experimental Botany 61: 199–223. 10.1016/j.envexpbot.2007.05.011.

[ece372939-bib-0044] Wang, C. , S. Jian , H. Ren , J. Yan , and N. Liu . 2021. “A Web‐Based Software Platform for Restoration‐Oriented Species Selection Based on Plant Functional Traits.” Frontiers in Ecology and Evolution 9: 570454. 10.3389/fevo.2021.570454.

[ece372939-bib-0045] Werden, L. K. , P. Alvarado , S. Zarges , et al. 2018. “Using Soil Amendments and Plant Functional Traits to Select Native Tropical Dry Forest Species for the Restoration of Degraded Vertisols.” Journal of Applied Ecology 55: 1019–1028. 10.1111/1365-2664.12998.

[ece372939-bib-0046] Westerband, A. C. , J. L. Funk , and K. E. Barton . 2021. “Intraspecific Trait Variation in Plants: A Renewed Focus on Its Role in Ecological Processes.” Annals of Botany 127: 397–410. 10.1093/aob/mcab011. 33507251 PMC7988520

[ece372939-bib-0047] Westerband, A. C. , A. K. Kagawa‐Viviani , K. K. Bogner , D. W. Beilman , T. M. Knight , and K. E. Barton . 2019. “Seedling Drought Tolerance and Functional Traits Vary in Response to the Timing of Water Availability in a Keystone Hawaiian Tree Species.” Plant Ecology 220: 321–344. 10.1007/s11258-019-00917-0.

[ece372939-bib-0048] Zhou, S.‐X. , B. E. Medlyn , and I. C. Prentice . 2015. “Long‐Term Water Stress Leads to Acclimation of Drought Sensitivity of Photosynthetic Capacity in Xeric but Not Riparian Eucalyptus Species.” Annals of Botany 117: 133–144. 10.1093/aob/mcv161. 26493470 PMC4701155

